# Effects of sodium-glucose cotransporter 2 inhibitors on cardiovascular and cerebrovascular diseases: a meta-analysis of controlled clinical trials

**DOI:** 10.3389/fendo.2024.1436217

**Published:** 2024-08-23

**Authors:** Fei Wang, Chunyu Li, Lili Cui, Shuo Gu, Junyu Zhao, Haipeng Wang

**Affiliations:** ^1^ Department of Endocrinology and Metabology, The First Affiliated Hospital of Shandong First Medical University & Shandong Provincial Qianfoshan Hospital, Shandong First Medical University, Shandong Key Laboratory of Rheumatic Disease and Translational medicine, Shandong Institute of Nephrology, Jinan, China; ^2^ School of Clinical Medicine, Jining Medical University, Jining, China; ^3^ Institute for Literature and Culture of Chinese Medicine, Shandong University of Traditional Chinese Medicine, Jinan, China; ^4^ Department of Radiology, Shandong Provincial Hospital Affiliated to Shandong First Medical University, Jinan, China

**Keywords:** sodium-glucose cotransporter 2 inhibitors, stroke, cardiovascular death, myocardial infarction, heart failure, all-cause mortality

## Abstract

**Objective:**

Evaluate the effects of sodium-glucose cotransporter 2 inhibitor (SGLT2i) on cardiovascular and cerebrovascular diseases.

**Methods:**

Articles of SGLT2i on cardiovascular and cerebrovascular diseases were searched. Two authors independently screened the literature, extracted the data, assessed the quality of the study and performed statistical analyses using Review Manager 5.4.

**Results:**

Random-effect model was used to merge the OR values, and the pooled effect showed that SGLT2i had significant preventive effects on cardiovascular death (OR=0.76, 95%CI 0.64 to 0.89), myocardial infarction (OR=0.90, 95%CI 0.84 to 0.96), heart failure (OR=0.69, 95%CI 0.64 to 0.74) and all-cause mortality (OR=0.65, 95%CI 0.58 to 0.73). Empagliflozin, dapagliflozin and canagliflozin all reduced the incidence of heart failure (OR=0.72, 95%CI 0.64 to 0.82; OR=0.56, 95%CI 0.39 to 0.80; OR=0.62, 95%CI 0.53 to 0.73), but only dapagliflozin displayed a favorable effect on inhibiting stroke (OR=0.78, 95%CI 0.63 to 0.98). SGLT2i could prevent stroke (OR=0.86, 95%CI 0.75 to 0.99), heart failure (OR=0.63, 95%CI 0.56 to 0.70) and all-cause mortality (OR=0.64, 95%CI 0.57 to 0.72) compared to DPP-4i. Furthermore, SGLT2i could reduce the incidence of heart failure (OR=0.72, 95%CI 0.67 to 0.77) and cardiovascular death (OR=0.72, 95%CI 0.54 to 0.95) in patients with high-risk factors.

**Conclusions:**

SGLT2i affects cardiovascular death, myocardial infarction, heart failure and all-cause mortality. Only dapagliflozin displayed a favorable effect on inhibiting stroke. SGLT2i could prevent stroke, heart failure and all-cause mortality compared to DPP-4i. In addition, SGLT2i significantly reduced the development of heart failure and cardiovascular death in patients with high-risk factors.

**Systematic review registration:**

https://www.crd.york.ac.uk/prospero, identifier CRD42024532783.

## 1 Introduction

Diabetes mellitus is a class of metabolic diseases characterized by hyperglycemia. Type 2 diabetes caused by relative insulin deficiency or insulin resistance is prevalent in clinical practice. With the rapid development of the socio-economic conditions, the prevalence of type 2 diabetes has shown an increasing trend with each passing year. According to the study, there will be more than 640 million people with type 2 diabetes in 2024 (1). Hyperglycemia is often associated with disorders of lipid and protein metabolism, which induces and exacerbates oxidative stress and increases the risk of atherosclerotic vascular disease. Patients are highly susceptible to adverse outcomes such as cardiovascular disease, stroke or chronic renal insufficiency if they do not receive effective treatment at an early age (2–5). Cardiopathy and stroke are second only to cancer in terms of death and disability; the hyperglycemic state of the body results in a poor prognosis for cardiovascular disease. Currently, there is a limited range of antihyperglycemic agents (AHAs) available in the clinic and multiple drug loads may have adverse effects on the liver or kidney. So, it is crucial to choose a safe and effective class of glucose-lowering drugs.

Sodium-glucose cotransporter 2 inhibitor (SGLT2i) is a class of prescription drugs approved for the treatment of type 2 diabetes. SGLT2i reduces blood glucose without increasing the risk of hypoglycemia in patients with type 2 diabetes by blocking glucose and sodium reabsorption in renal proximal tubules (6). In addition, the mechanism of promoting urinary sodium excretion and diuresis by SGLT2i may allow it to decrease blood pressure and weight without increasing the heart rate, which has a preventive effect on the progression of atherosclerotic heart disease, heart failure or chronic kidney disease (6–9). Some findings suggested that SGLT2i could reduce the risk of stroke in Asian patients with type 2 diabetes (10); Zhou speculated that this favorable effect may be related to the reduction of atrial fibrillation/atrial flutter by SGLT2i (11). A meta-analysis found that although SGLT2i was more appropriate for type 2 diabetes patients who were at high risk of stroke compared to dipeptidyl peptidase 4 inhibitor (DPP-4i), the results of this study showed that SGLT2i did not reduce the risk of stroke (12). Therefore, we need to confirm the cardiovascular and cerebrovascular effects of SGLT2i in further clinical studies as well as to verify whether the effect is related to diseases or race/ethnicity.

Up to now, several clinical studies have reported the therapeutic effects of SGLT2i on cardiovascular and cerebrovascular diseases (10, 13–60); but the evidence needs to deepen due to the differences in search strategies, interventions, inclusion populations, sample sizes and other factors. In this study, we conducted a meta-analysis of clinical controlled trials on cardiovascular and cerebrovascular diseases with SGLT2i by systematically searching literature at home and abroad.

## 2 Materials and methods

### 2.1 Searching progress

We searched of the following databases: PubMed, Cochrane library and Sinomed for clinical controlled trials of SGLT2i on the effects of cardiovascular and cerebrovascular diseases. Reference lists of all eligible articles and related previous review articles were also manually searched. The literature search for this meta-analysis was restricted to published results. Databases were searched from the earliest data to 3 January 2024 with the search terms: ((SGLT2 inhibitors) OR (Sodium-Glucose Transporter 2 Inhibitors) OR (Sodium-glucose cotransporter-2 inhibitors) OR (Dapagliflozin) OR (Canagliflozin) OR (Empagliflozin) OR (Ipragliflozin) OR (Luseogliflozin) OR (Tofogliflozin)) AND ((acute cerebral infarction) OR (acute cerebral stroke) OR (ischemia stroke) OR (cerebral infarction)) AND ((cardiac failure) OR (acute cardiac failure) OR (heart failure) OR (acute heart failure) OR (cardiac insufficiency) OR (congestive cardiac failure) OR (congestive heart failure)) AND ((myocardial infarction) OR (acute myocardial infarction) OR (ST-segment elevation myocardial infarction) OR (ST elevated acute myocardial infarction) OR (non-ST-elevation myocardial infarction) OR (heart attack)).

Eligible studies were screened and selected based on the following criteria: (1) published in English or Chinese language; (2) evaluated the effect of SGLT2i intervention in cardiovascular and cerebrovascular diseases; (3) clinical controlled trial; (4) reported at least one outcome.

### 2.2 Study selection and data extraction

Two reviewers independently checked all titles and abstracts for studies that could potentially meet the inclusion criteria. We retrieved full reports of these potentially eligible studies for detailed assessment by two reviewers, who then independently extracted information on study design, drug use, study location, characteristics of participants, sample size and relevant outcomes on to a preformatted spreadsheet (10, 13–60). Any uncertainties or discrepancies between the two reviewers were resolved through consensus after rechecking of the source data and consultation with the third reviewer. We also contacted authors if any areas of uncertainty needed clarification.

### 2.3 Risk of bias in results of included studies

Two reviewers independently assessed the risk of bias in included studies to avoid conflicts of interest of study investigators or funders. Randomized controlled trials (RCTs) were evaluated using the revised version of the Cochrane tool, known as RoB 2. While cohort studies were evaluated using the Risk Of Bias In Non-randomized Studies of Interventions (ROBINS-I) tool, which is recommended for assessing risk of bias in a non-randomized study of interventions (NRSI) (61, 62). The articles were evaluated separately by two reviewers and disagreements were settled by discussion.

### 2.4 Statistical analysis

The primary outcomes were the incident rate of stroke, cardiovascular death, myocardial infarction, heart failure or all-cause mortality. The secondary outcomes were the incident rate of ischemic stroke, acute coronary syndrome (ACS) or revascularization. Subgroup analyses were carried out according to differences in interventions and population characteristics. The fixed-model was performed by odds ratio (OR) and 95% confidence intervals (CI) for dichotomous variables. The I^2^ was calculated as an index of heterogeneity between studies. If a considerable heterogeneity exists, then the fixed-effects model is replaced by the random-effect model. The analyses were performed by Review Manager 5.4 (Cochrane Collaboration, United Kingdom, http://www.cochrane.org).

## 3 Results

### 3.1 Search results and characteristics of included studies

**Figure 1:**
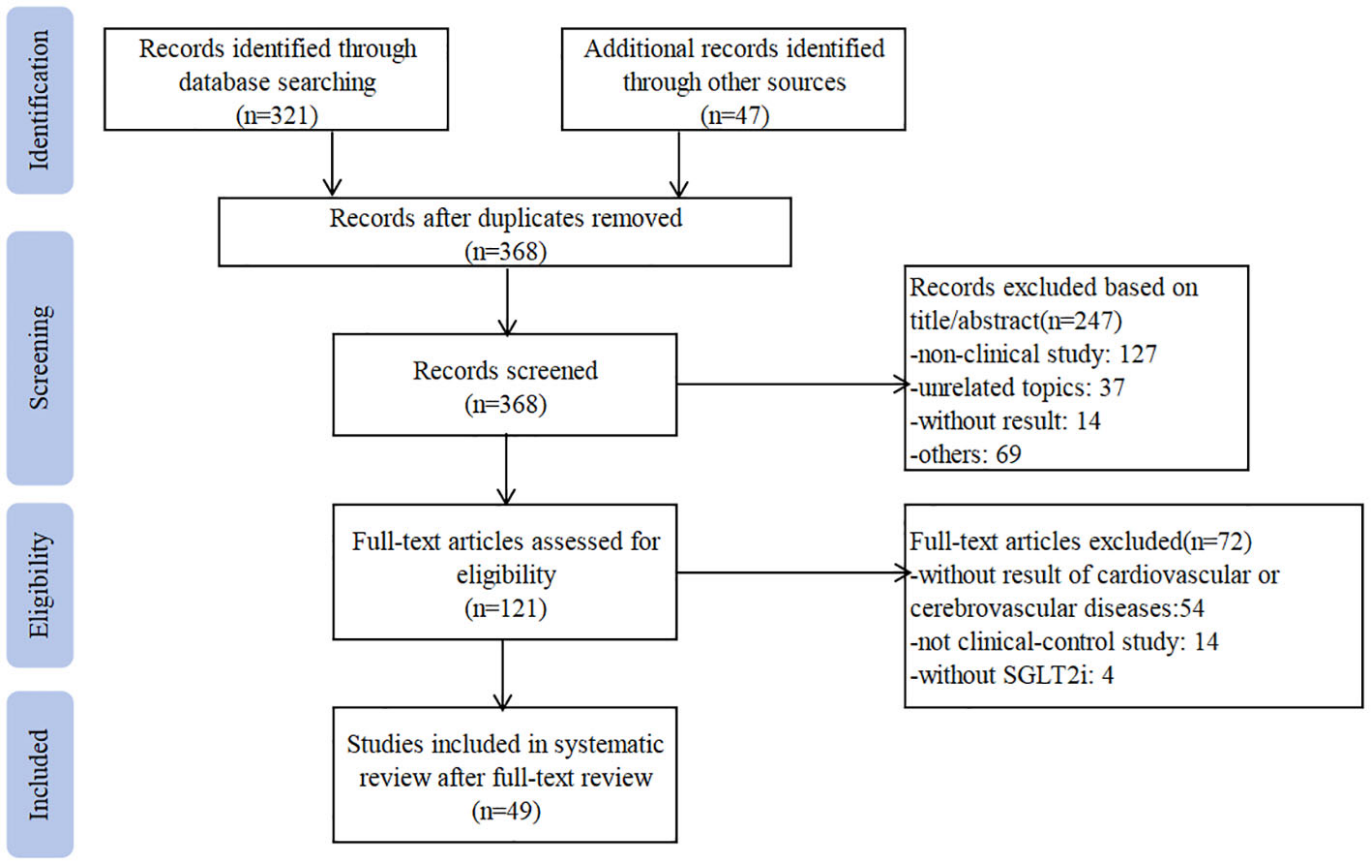
Flow chart of the systematic search process.

Our research yielded 368 articles in English or Chinese that were potentially relevant to this study. After screening the abstract, 121 articles were selected for full-text review. Of these, 49 articles were eligible and included in this meta-analysis (10, 13–60). Searching progress is shown in **Figure 1**. Nine of the included studies were RCT (14, 23, 40, 42–45, 48, 60), and the rest 40 trials were cohort studies. Nine trials were multi-center studies (14, 16, 23, 24, 31, 36, 40, 42, 43). Eight studies were published in Chinese (44–49, 59, 60), and the rest were published in English. There are 1270038 patients received SGLT2i treatment (dapagliflozin: 21145 patients (27, 36, 40, 41, 46, 47, 49, 60); empagliflozin: 110227 patients (20, 23, 27, 32, 39, 42, 43); canagliflozin:55950 patients (33, 44, 45, 48, 59) and 1339802 assigned to the control group (glucagon-like peptide 1 (GLP-1RA): 427963 patients (15, 17, 20, 28, 30, 33–35, 37, 39, 53); DPP-4i: 469049 patients (10, 18, 20–22, 26, 27, 31–33, 36, 50, 53, 55–58). The sample size ranges from 30 to 133139 in the SGLT2i treatment group and the control group. Due to the large sample size and complex population characteristics of this meta-analysis, the exact dosage and frequency of treatment regimens were unclear. Only eleven trials reported the precise time of follow-up, and ranged from 1 month to 2 years (16, 20, 29, 37, 44, 45, 48–50, 56, 60). The detailed characteristics of the included studies are summarized in **Supplementary Table 1**.

### 3.2 Risk of bias

**Figure 2:**
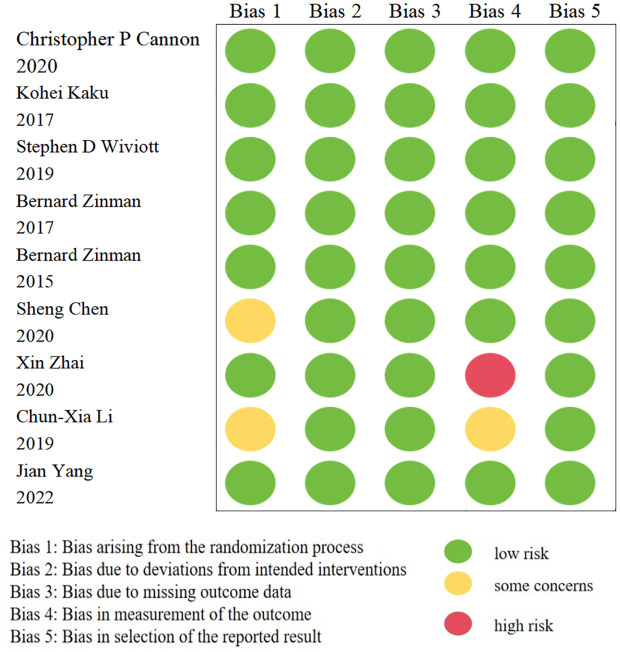
Risk of bias in RCTs.

**Figure 3:**
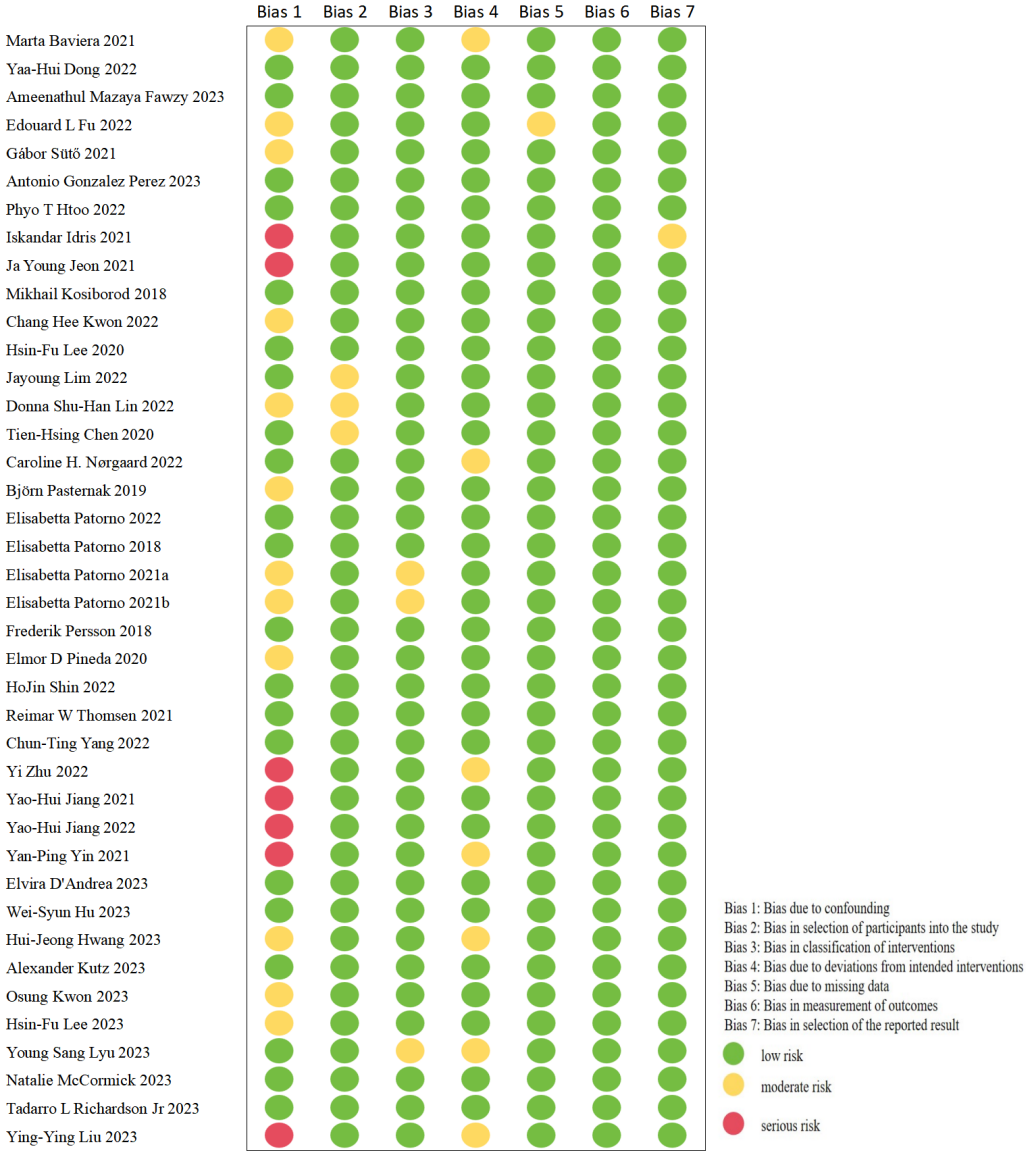
Risk of bias in cohort studies.

In this meta-analysis, only nine trials were RCTs (14, 23, 40, 42–45, 48, 60); one of which was found to be a high-risk trial after evaluating the quality of these studies with RoB 2 tool (45). The details are illustrated in **Figure 2**. The remaining cohort studies were evaluated in 7 dimensions for risk of bias using the ROBINS-I tool (10, 13, 15–22, 24–39, 41, 46, 47, 49–59). **Figure 3** shows that 17 (42.5%) of the 40 papers had a low risk of bias (10, 15, 16, 19, 20, 24, 26, 32, 33, 36, 38, 39, 50, 51, 53, 57, 58), 16 (40%) had a medium risk of bias (13, 17, 18, 25, 27–31, 34, 35, 37, 52, 54–56) and 7 (17.5%) had a high risk of bias (21, 22, 41, 46, 47, 49, 59).

### 3.3 Main outcome

#### 3.3.1 Incidence of stroke

**Figure 4:**
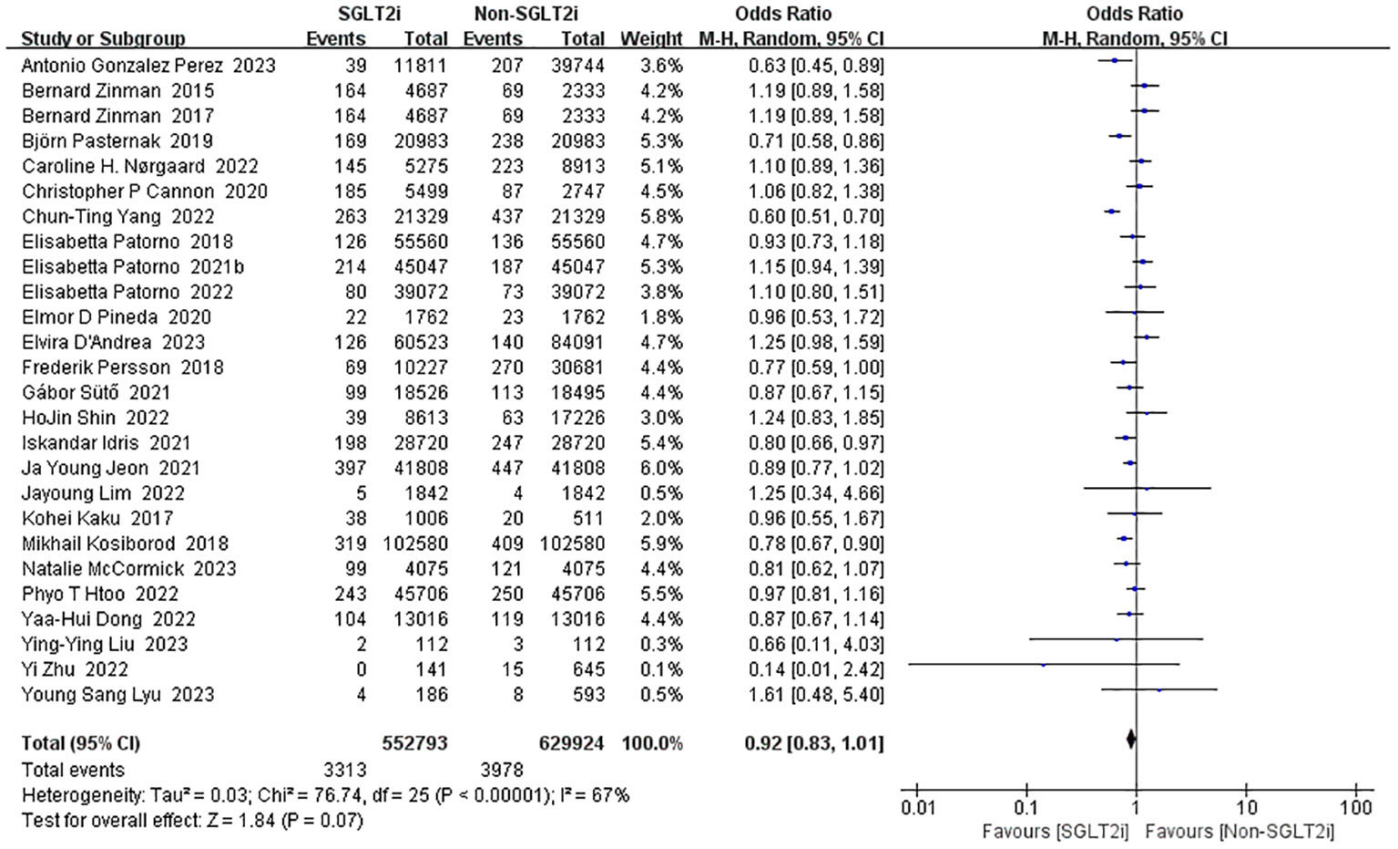
Forest plot of the incidence of stroke.

Of these 49 included studies, 26 studies of SGLT2i with other AHAs reported the rate of incidence of stroke as an outcome (10, 14, 15, 18–24, 27, 30–33, 35–38, 41–43, 50, 56, 57, 59). A fixed-effect model was used for the pooled effect of these studies, which showed a significant heterogeneity (heterozygosity test, Chi^2^ = 76.74, *P*<0.00001, *I^2^* = 67%). Then, we used the random-effect model for comparison, which showed that SGLT2i did not reduce the incidence of stroke (OR=0.92, 95%CI 0.83 to 1.01, *P*=0.07) (**Figure 4**).

#### 3.3.2 Incidence of cardiovascular death

**Figure 5:**
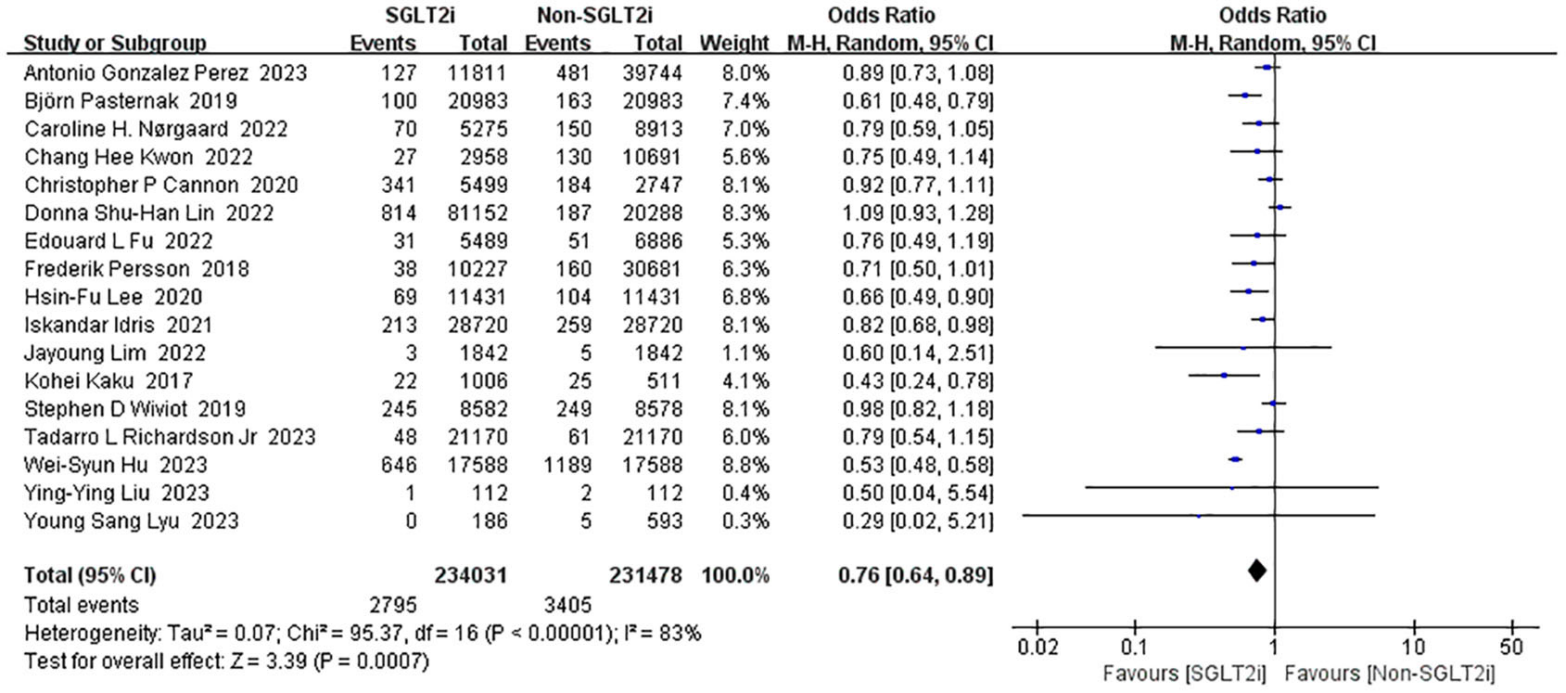
Forest plot of the incidence of cardiovascular death.

Seventeen studies reported the effect of SGLT2i intervention on cardiovascular death (14, 17, 19, 21, 23, 25–28, 30, 31, 36, 40, 51, 56, 58, 59). Analyses using the fixed-effect model showed enormous heterogeneity (heterozygosity test, Chi^2^ = 95.37, *P*<0.00001, *I^2^* = 83%). So, the studies were instead analyzed using random-effect model and the merged OR value of the effect value was 0.76 (95%CI 0.64 to 0.89, *P*=0.0007) (**Figure 5**). Thus, the SGLT2i treatment group reduced the incidence of cardiovascular death compared to the non-SGLT2i control group.

#### 3.3.3 Incidence of myocardial infarction

**Figure 6:**
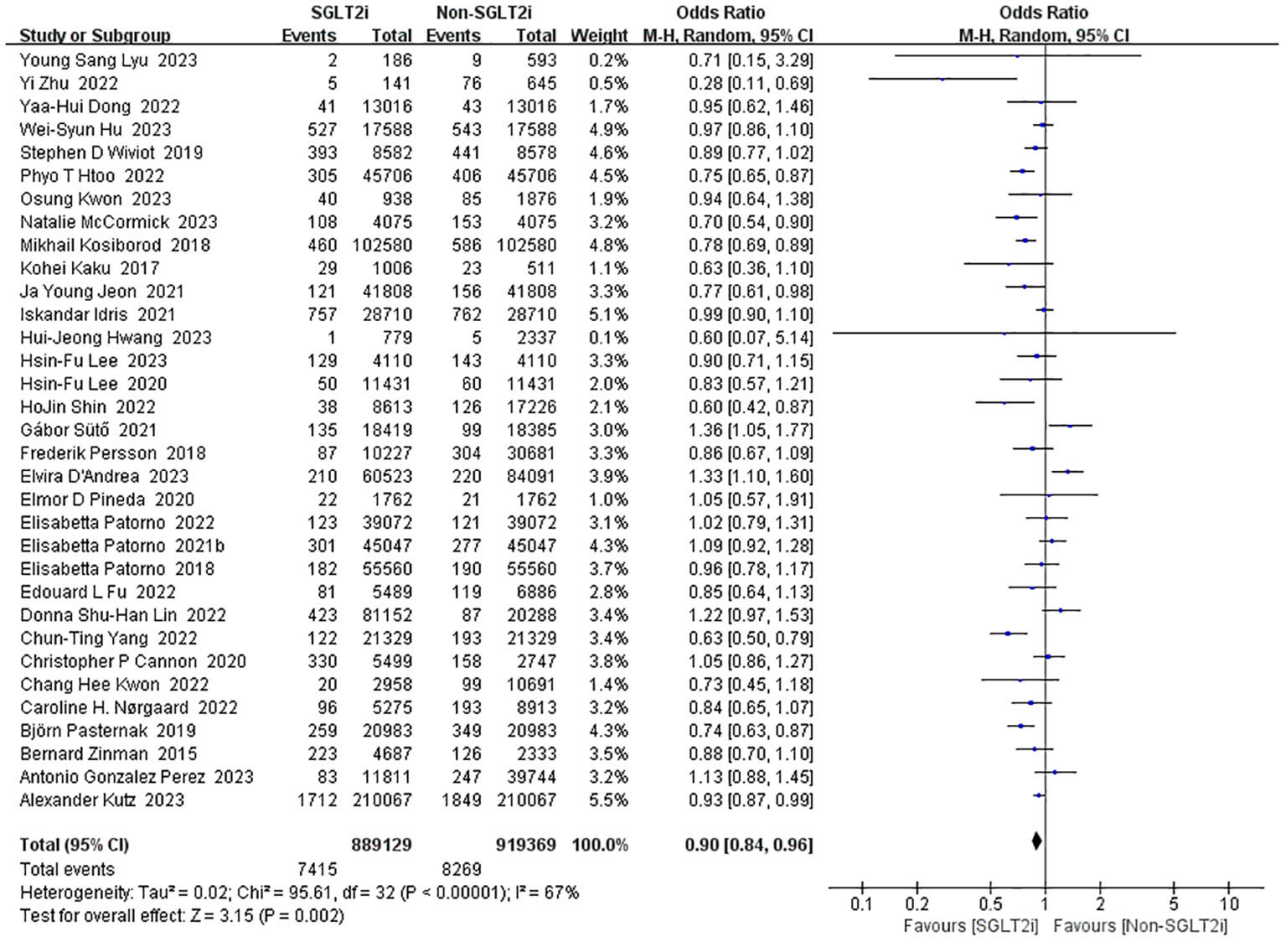
Forest plot of the incidence of myocardial Infarction.

A total of 33 studies reported the incidence of myocardial infarction with SGLT2i intervention (10, 14, 15, 17–26, 28, 30–33, 35–38, 40, 41, 43, 50–57). Heterogeneity was significant in the fixed-effect model analysis of these studies (heterozygosity test, Chi^2^ = 95.61, *P*<0.00001, *I^2^* = 67%), after that, a random-effect model was used and pooled effect value was 0.90 (95%CI 0.84 to 0.96, *P*=0.002) (**Figure 6**). Thus, SGLT2i could reduce the incidence of myocardial infarction.

#### 3.3.4 Incidence of heart failure

**Figure 7:**
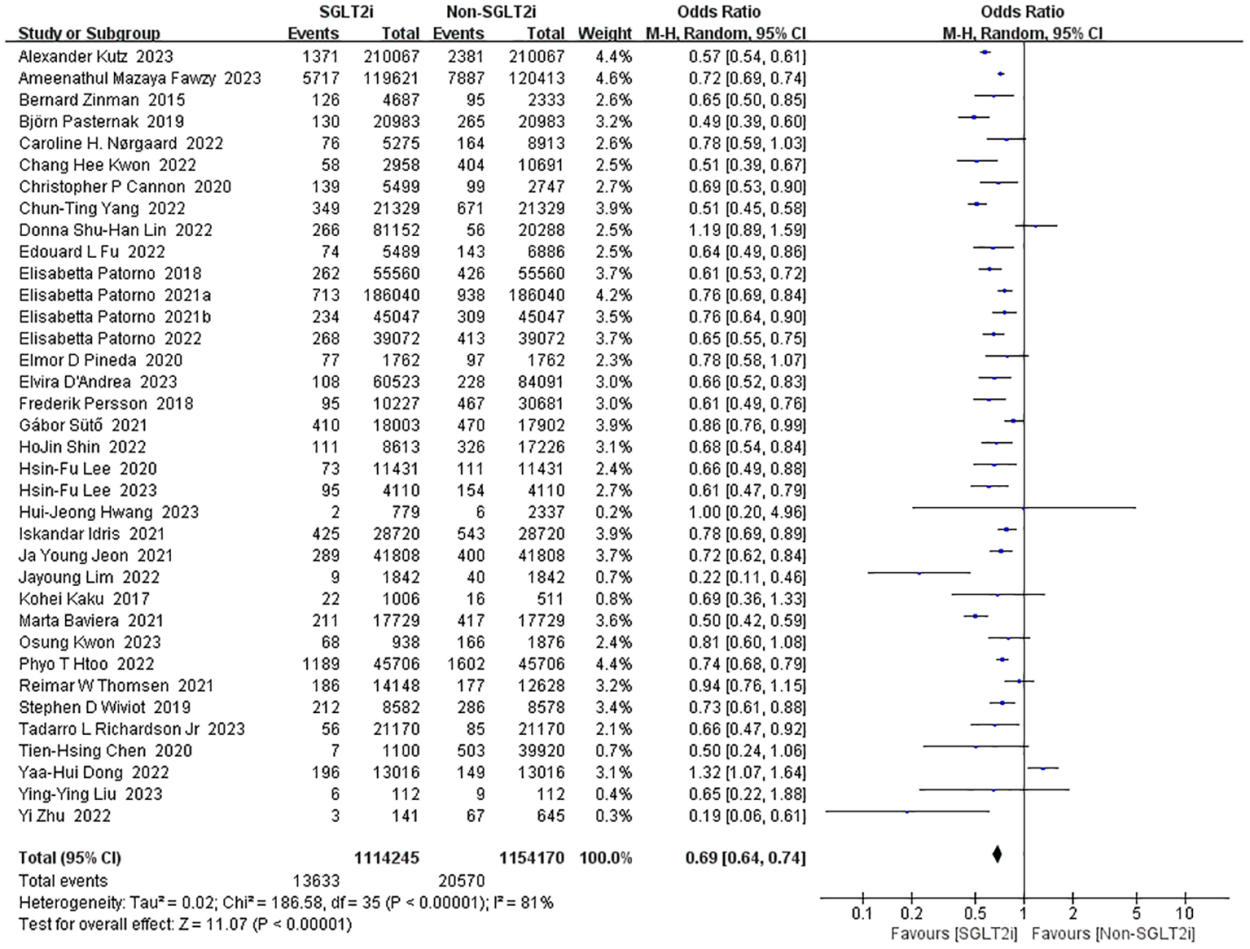
Forest plot of the incidence of heart failure.

36 studies reported the incidence of heart failure (10, 13–18, 20–23, 25–41, 43, 50, 52–55, 58, 59). Heterogeneity analysis of these studies showed substantial heterogeneity (heterozygosity test, Chi^2^ = 186.58, *P*<0.00001, *I^2^* = 81%). Therefore, the analysis was performed using the random-effect model with a pooled effect value of 0.69 (95%CI 0.64 to 0.74, *P*<0.00001) (**Figure 7**). It can be indicated that SGLT2i significantly reduced the occurrence of heart failure compared with non-SGLT2i.

#### 3.3.5 Incidence of all-cause mortality

**Figure 8:**
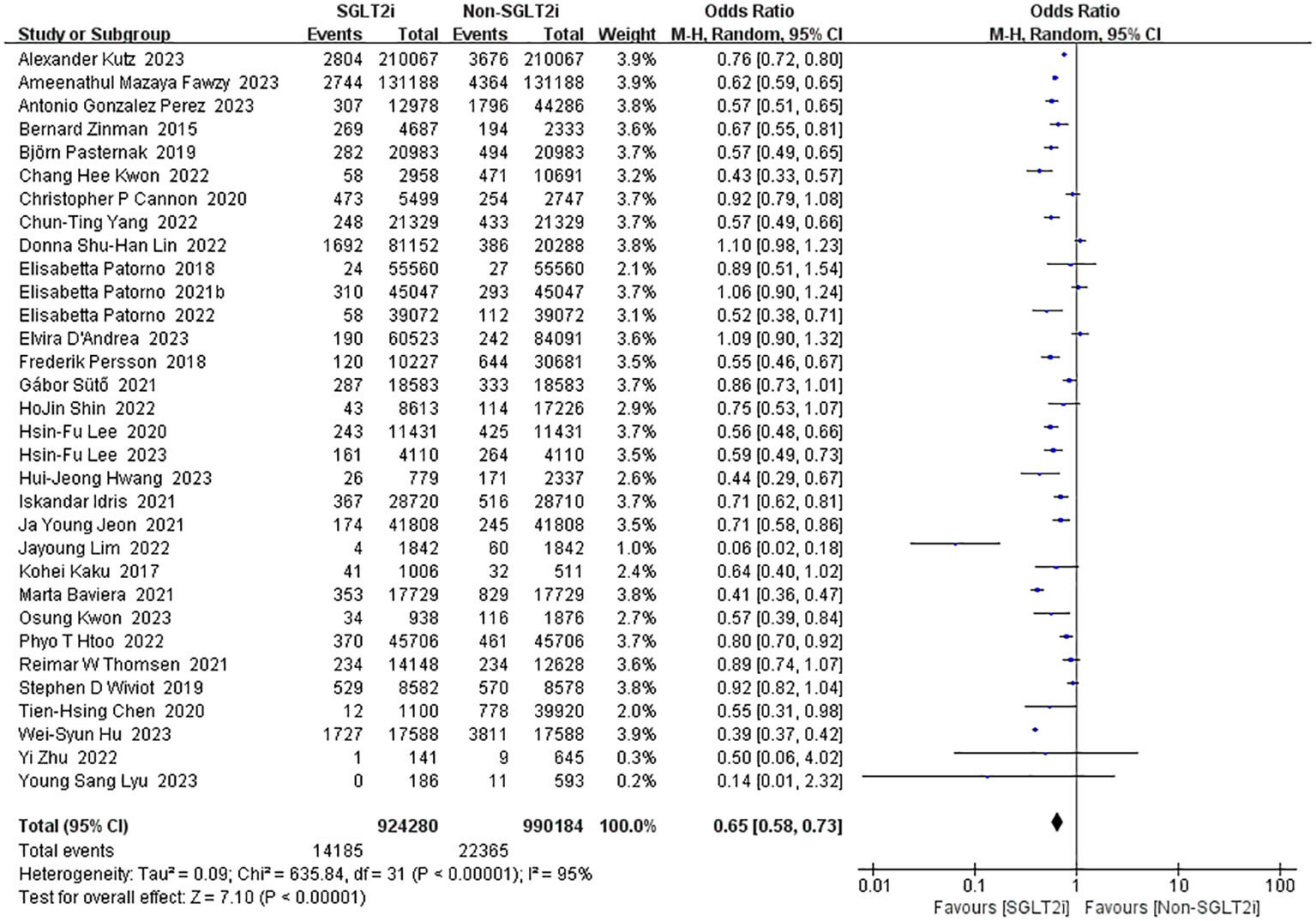
Forest plot of the incidence of all-cause mortality.

Among the intervention studies of SGLT2i, 32 studies reported all-cause mortality (10, 13, 14, 16, 18–23, 25–29, 31–33, 35, 36, 38–41, 43, 50–56). As there was substantial heterogeneity (heterozygosity test, Chi^2^ = 635.84, *P*<0.00001, *I^2^* = 95%), pooled analyses using the random-effect model was instead which resulted in a favorable pooled effect value of 0.65 (95%CI 0.58 to 0.73, *P*<0.00001) for SGLT2i (**Figure 8**). In summary, SGLT2i could reduce all-cause mortality and improve survival.

### 3.4 Secondary outcome

#### 3.4.1 Incidence of ischemic stroke

Fourteen studies reported the incidence of ischemic stroke between SGLT2i group and non-SGLT2i group (13, 15–17, 25, 26, 28, 29, 40, 51–55). Firstly, we pooled the OR value from these studies by fixed-effect model, as a result, a significant heterogeneity was found (heterozygosity test, Chi^2^ = 47.65, *P*<0.00001, *I^2^* = 73%). Then the random-effect model was instead, and it was found that SGLT2i could not reduce ischemic stroke in patients (OR=0.95, 95%CI 0.87 to 1.05, *P*=0.32).

#### 3.4.2 Incidence of revascularization

Only six studies reported the incidence of revascularization as an outcome (27, 33, 37, 43, 55, 56). A pooled analysis of outcome events from these studies using the fixed-effect model revealed tremendous heterogeneity of results (heterozygosity test, Chi^2^ = 23.96, *P*=0.0002, *I^2^* = 79%). When analyzed using the random-effect model, the merged OR value was 0.85 (95%CI 0.65 to 1.11, *P*=0.23). In conclusion, SGLT2i did not reduce the occurrence of revascularization.

#### 3.4.3 Incidence of acute coronary syndrome

**Figure 9:**
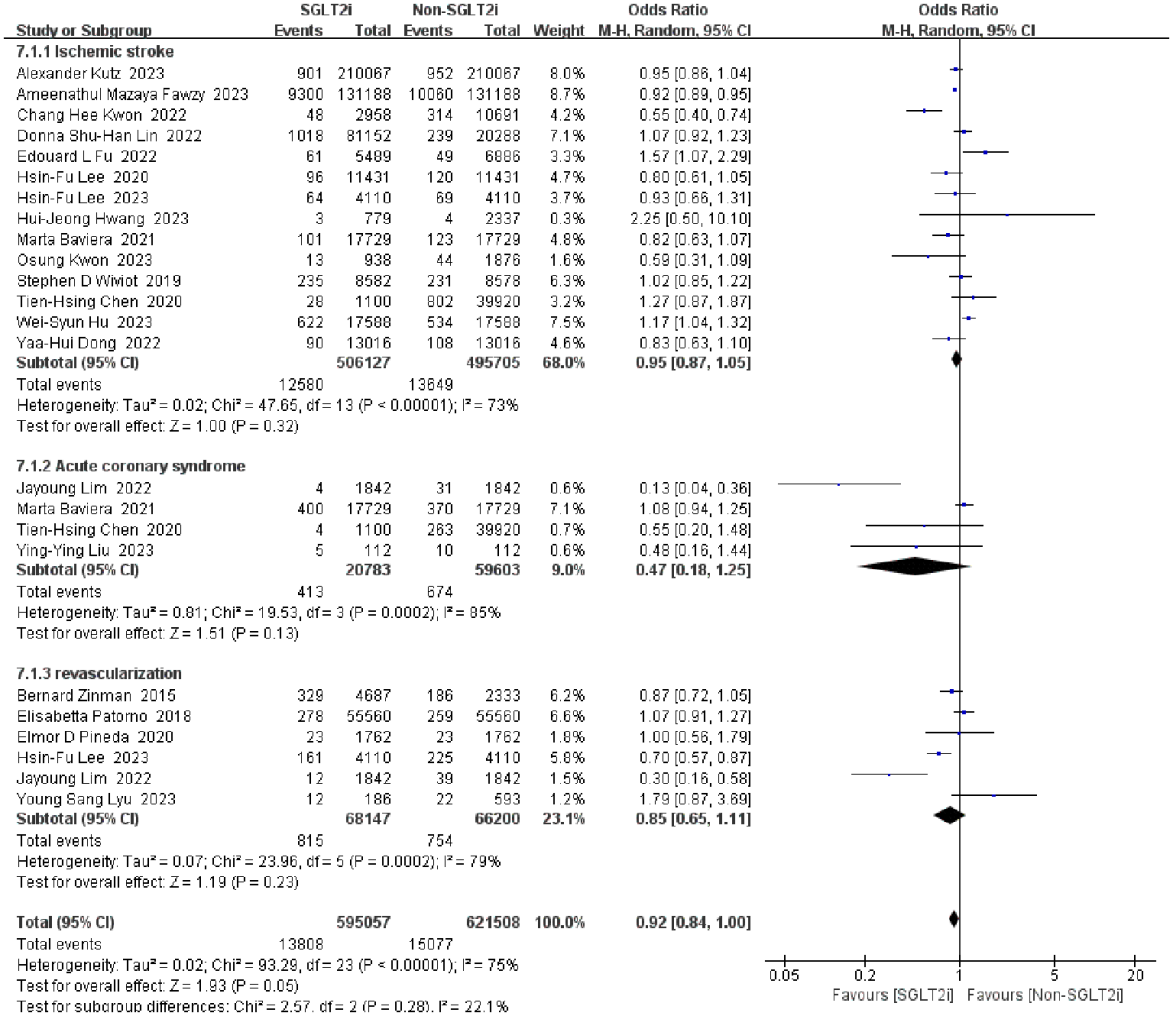
Forest plot of the incidence of secondary outcomes (ischemic stroke, revascularization, ACS).

Events of ACS in patients with SGLT2i have been reported in four studies (13, 27, 29, 59). However, a significantly and huge heterogeneity was found by fixed-effect model (heterozygosity test, Chi^2^ = 19.53, *P*=0.0002, *I^2^* = 85%), then a random-effect model was used and pooled OR value was 0.98(95%CI 0.86 to 1.12, *P*=0.77). This means that SGLT2i is not beneficial in preventing the development of ACS. Detailed data for the above secondary endpoints are shown in **Figure 9**.

### 3.5 Subgroup analyses

#### 3.5.1 Incidence of cardiovascular and cerebrovascular diseases under different interventions

Seven studies analyzed the effect of empagliflozin on cardiovascular and cerebrovascular diseases (20, 23, 27, 32, 39, 42, 43); eight studies analyzed the effect of dapagliflozin on it (27, 36, 40, 41, 46, 47, 49, 60); and only five studies analyzed the effect of canagliflozin on it (33, 44, 45, 48, 59). Appropriate effect models were selected based on the magnitude of heterogeneity. Pooling these studies about different types of SGLT2i revealed that dapagliflozin prevented stroke (OR=0.78, 95%CI 0.63 to 0.98, *P*=0.03), myocardial infarction (OR=0.83, 95%CI 0.74 to 0.93, *P*=0.002), heart failure (OR=0.56, 95%CI 0.39 to 0.80, *P*=0.002), and all-cause mortality (OR=0.50, 95%CI 0.30 to 0.82, *P*=0.006). At the same time, empagliflozin reduced the incidence of myocardial infarction (OR=0.82, 95%CI 0.73 to 0.91, *P*=0.0003), heart failure (OR=0.72, 95% CI 0.64 to 0.82, *P*<0.00001), and all-cause mortality (OR=0.68, 95%CI 0.55 to 0.84, *P*=0.0004); canagliflozin only had a positive effect on the occurrence of heart failure (OR=0.56, 95%CI 0.39 to 0.80, *P*=0.002).

Eleven studies reported the therapeutic effects of SGLT2i on cardiovascular and cerebrovascular diseases compared with GLP-1RA. These studies reported four diseases (including stroke, myocardial infarction, heart failure and all-cause mortality) and the details on the occurrence of each disease were provided (15, 17, 20, 28, 30, 33–35, 37, 39, 53). It was found that SGLT2i only had a significant preventive effect on heart failure (OR=0.83, 95%CI 0.74 to 0.93, *P*=0.002) compared to GLP-1RA.

Four diseases (including stroke, myocardial infarction, heart failure and all-cause mortality) were reported in seventeen studies (10, 18, 20–22, 26, 27, 31–33, 36, 50, 53, 55–58). What a pity, a considerable heterogeneity was found in all four subgroups by the fixed-effect model. Finally, a random-effect model was used and pooled OR value was 0.86 (95%CI 0.75 to 0.99, *P*=0.04) in the subset of stroke, 0.63 (95%CI 0.56 to 0.70, *P*<0.00001) in the subset of heart failure, and 0.64 (95%CI 0.57 to 0.72, *P*<0.00001) in the subset of all-cause mortality.

The details of the above are shown in **Table 1**. Summarily, in different types of SGLT2i, empagliflozin, dapagliflozin and canagliflozin all reduced the incident rate of heart failure, but only dapagliflozin could reduce the incident rate of stroke. Compared with DPP-4i, SGLT2i had a positive therapeutic effect on stroke, heart failure and all-cause mortality; however, compared with GLP-1RA, it only had a positive impact on heart failure.

**Table 1:** The incidence of cardiovascular and cerebrovascular diseases in different intervention measures.

Outcomes of different interventions	Sample size		OR	95%CI	P	Heterogeneity		Model
	Intervention	Control				I2 (%)	P	
Empagliflozin vs Non-Empagliflozin								
stroke	96079	92718	1.06	0.94,1.20	0.33	0	0.79	Fixed
Myocardial infarction	90471	87622	0.82	0.73,0.91	0.0003	44	0.15	Fixed
Heart failure	105540	103013	0.72	0.64,0.82	<0.00001	52	0.07	Random
All-cause mortality	105540	103013	0.68	0.55,0.84	0.0004	75	0.001	Random
Dapagliflozin vs Non-Dapagliflozin								
stroke	12561	36529	0.78	0.63,0.98	0.03	0	0.77	Fixed
Myocardial infarction	20222	42344	0.83	0.74,0.93	0.002	35	0.16	Fixed
Heart failure	19920	42715	0.56	0.39,0.80	0.002	66	0.02	Random
All-cause mortality	20393	43719	0.50	0.30,0.82	0.006	88	<0.00001	Random
Canagliflozin vs Non-Canagliflozin								
stroke	55750	55750	0.91	0.72,1.16	0.46	0	0.83	Fixed
Myocardial infarction	55756	55755	0.94	0.77,1.15	0.54	0	0.72	Fixed
Heart failure	55868	55867	0.62	0.53,0.73	<0.00001	0	0.98	Fixed
All-cause mortality	55756	55755	0.40	0.13,1.28	0.12	67	0.05	Random
SGLT2i vs GLP-1RA								
stroke	107718	111356	1.09	0.97,1.21	0.14	0	0.65	Fixed
Myocardial infarction	284224	228395	0.98	0.91,1.05	0.54	19	0.28	Fixed
Heart failure	484412	427063	0.83	0.74,0.93	0.002	79	<0.00001	Random
All-cause mortality	273645	211261	1.00	0.94,1.05	0.90	29	0.22	Fixed
SGLT2i vs DPP-4i								
stroke	267650	312048	0.86	0.75,0.99	0.04	72	<0.0001	Random
Myocardial infarction	401444	445839	0.89	0.80,1.00	0.04	76	<0.00001	Random
Heart failure	419779	463700	0.63	0.56,0.70	<0.00001	84	<0.00001	Random
All-cause mortality	399375	443804	0.64	0.57,0,72	<0.00001	83	<0.00001	Random

#### 3.5.2 Incidence of cardiovascular and cerebrovascular diseases in different characteristics of patients

Furthermore, fifteen studies explicitly stated whether the subjects had cardiovascular and cerebrovascular diseases or were at other high risk (14, 15, 21, 23, 25, 27, 34, 40, 42, 43, 51, 54–56, 59). Firstly, four outcomes (including stroke, myocardial infarction, heart failure and cardiovascular death) in these studies were analyzed by using the fixed-effect model. However, some significantly and huge heterogeneity were found, then appropriate effect models were selected based on the magnitude of heterogeneity. It was found that SGLT2i demonstrated significant benefits in heart failure (OR=0.72, 95%CI 0.67 to 0.77, *P*<0.00001) and cardiovascular death (OR=0.72, 95%CI 0.54 to 0.95, *P*=0.02) in high-risk patients (**Table 2**).

**Table 2:** The incidence of cardiovascular and cerebrovascular diseases in different characteristics of patients.

Outcomes of different population characteristics	Sample size		OR	95%CI	P	Heterogeneity		Model
	Intervention	Control				I2 (%)	P	
with cardiovascular and cerebrovascular risk factors								
stroke	27358	19403	1.00	0.88,1.13	0.95	44	0.11	Fixed
Myocardial infarction	56921	60394	0.95	0.89,1.01	0.08	0	0.63	Fixed
Heart failure	92160	95226	0.72	0.67,0.77	<0.00001	17	0.29	Fixed
Cardiovascular death	47298	52187	0.72	0.54,0.95	0.02	88	<0.00001	Random
without cardiovascular and cerebrovascular risk factors								
stroke	32211	32211	0.89	0.73,1.09	0.26	0	0.87	Fixed
Myocardial infarction	30369	30369	0.94	0.76,1.17	0.58	0	0.95	Fixed
Heart failure	165350	165350	0.73	0.47,1.13	0.15	<0.00001	90	Random
Cardiovascular death	19195	19195	0.85	0.61,1.19	0.35	0	0.62	Fixed

### 3.6 Publication bias

**Figure 10:**
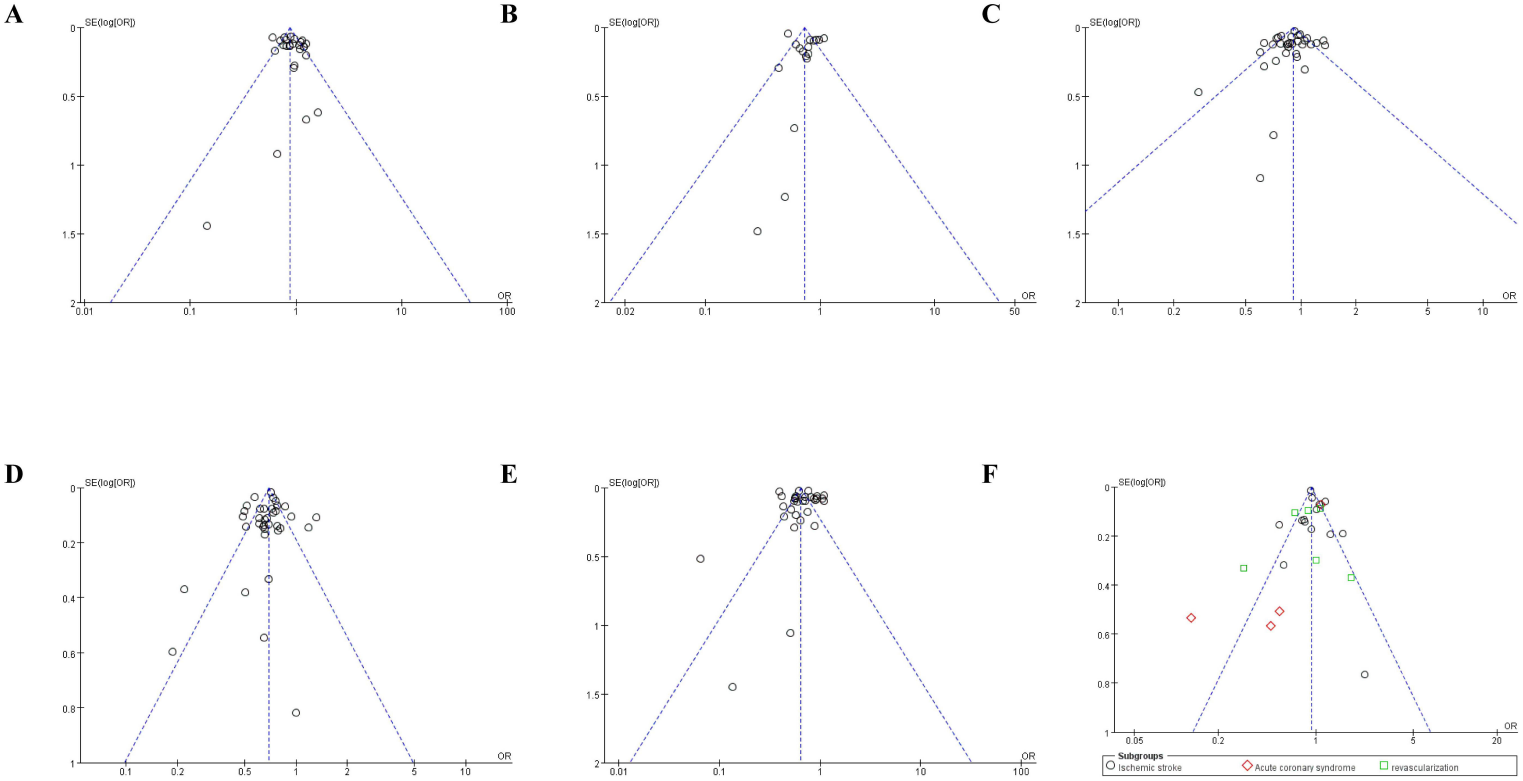
Funnel plot of publication bias on main and secondary outcomes. **(A)** Funnel plot of publication bias on stroke; **(B)** Funnel plot of publication bias on cardiovascular death; **(C)** Funnel plot of publication bias on myocardial Infarction; **(D)** Funnel plot of publication bias on heart failure; **(E)** Funnel plot of publication bias on all-cause mortality; **(F)** Funnel plot of publication bias on secondary outcomes.

**Figure 11:**
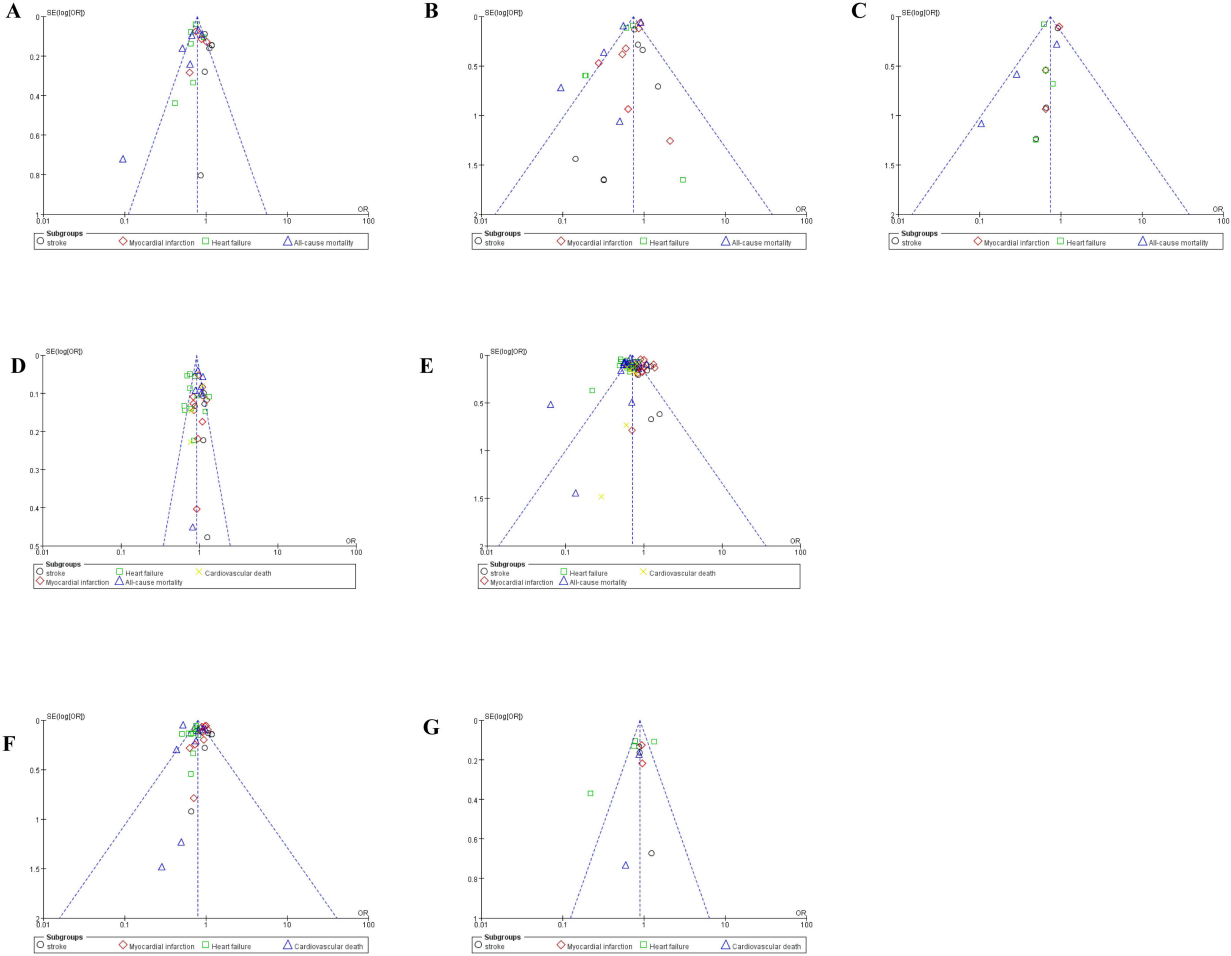
Funnel plot of publication bias on subgroup analysis. **(A)** Funnel plot of publication bias on subgroup analysis of Empagliflozin; **(B)** Funnel plot of publication bias on subgroup analysis of Empagliflozin Dapagliflozin; **(C)** Funnel plot of publication bias on subgroup analysis of Canagliflozin; **(D)** Funnel plot of publication bias on subgroup analysis of SGLT2i VS GLP-1RA; **(E)** Funnel plot of publication bias on subgroup analysis of SGLT2i VS DPP-4i; **(F)** Funnel plot of publication bias on subgroup analysis who were at high risk; **(G)** Funnel plot of publication bias on subgroup analysis who were not at high risk.

Funnel plot was done to show the publication bias and results were shown in **Figures 10**, **11**. Because of the complexity of population characteristics included in the study and the large gaps in sample sizes, some of the graphs show asymmetry; that is, there is publication bias.

## 4 Discussion

SGLT2i is a new class of insulin-independent drug for type 2 diabetes, which acts highly selectively on renal proximal tubules to block glucose reabsorption and increase the elimination of excess glucose from the body (63). In order to clarify the intervention effect of SGLT2i on cardiovascular and cerebrovascular diseases, researchers have prepared and conducted several clinical trials. EMPA-REG OUTCOME was a multi-center prospective study in which investigators found that the empagliflozin group could significantly reduce the risk of major adverse cardiovascular events in type 2 diabetes patients who were at high risk compared to the placebo group after following up for mean 3.1 years (23). This finding eventually caused SGLT2i was recommended by the American Diabetes Association and the European Association for the Study of Diabetes for the treatment of high-risk type 2 diabetes patients who suffer from arteriosclerotic cardiovascular disease (64). Current studies have found that SGLT2i does not reduce the incidence of stroke (42), and to some extent, it even increases the risk of ischemic stroke (65). The results of our study showed that SGLT2i does have great advantages in the prevention of cardiovascular and cerebrovascular diseases: SGLT2i could significantly reduce the incidence of myocardial infarction, heart failure, cardiovascular death and all-cause mortality; in subgroup analyses, the risk of heart failure was seen to be decreased by SGLT2i regardless of the type of SGLT2i; furthermore, in high-risk patients, SGLT2i exerted a positive effect in preventing the occurrence of heart failure and cardiovascular death. It was interesting to note that although SGLT2i reduced the risk of stroke compared to DPP-4i, but it had no preventative effect on the occurrence of stroke or ischemic stroke when comparing to non-SGLT2i.

At present, the mechanism of SGLT2i intervention in cardiovascular and cerebrovascular diseases is still being discovered and improved. Hemodynamic optimization and renal effect were thought to be the main two mechanisms (23, 66). Osmotic diuresis by SGLT2i reduces blood volume and cardiac load; in turn, sodium excretion decreases intraglomerular pressure by activating tubuloglomerular feedback (23, 67). On the other hand, SGLT2i has been shown to enhance endothelial cell function by reducing inflammation and oxidative stress (41, 68, 69), thereby improving coronary blood flow and myocardial energy metabolism (70). In contrast, SGLT2i is not as effective for stroke. Hypovolemia and elevated hematocrit from osmotic diuresis may be associated with an increased risk of stroke (42, 43), which seems to be a plausible explanation given that a meta-analysis has found that upright hypotension increases the risk of stroke (71).

GLP-1RA enhances insulin secretion by activating the GLP-1 receptor and inhibits glucagon secretion. It is able to delay gastric emptying and reduce the amount of food intake through central appetite suppression, ultimately achieving the effects of lowering blood glucose and body weight (72). In recent years, with the in-depth studies of the drug, researchers have found that in addition to its hypoglycemic and weight-loss effects, it can improve mitochondrial dysfunction, reduce inflammatory mediators and leukocyte-endothelial interactions, which can prevent the onset and progression of atherosclerosis (73). DPP-4i promotes insulin release from pancreatic beta cells by reducing the inactivation of glucagon-producing polypeptide (74). A large number of studies have been conducted on the comparative clinical efficacy of these three classes of drugs, but the conclusions are conflicting (13, 17, 22, 30, 34, 36, 75). In addition, studies have found that empagliflozin improves sympathetic nerve activity and is more favorable for glycemic control and management of cardiometabolic parameters (76, 77); while dapagliflozin shows more benefits in heart failure (78, 79). In previous studies, investigators have found some heterogeneity in outcome comparisons, which depending on the presence of chronic cardiac and renal diseases in patients before inclusion in the study. With the above in mind, this study conducted a number of subgroup analyses to further analyze the clinical effects of SGLT2i from multiple perspectives.

Although a large number of articles have been published on the topic of SGLT2i and cardiovascular diseases, there are some unique aspects of our work. In this study, we added the keyword “stroke” to focus more on the cerebrovascular diseases which are controversial. We included more studies and larger sample than others, and got more results, what is a supplement to the previous meta-analysis. Patients with type 2 diabetes often have multiple comorbidities, such as microvascular disease and renal disease, which has led to high-risk bias when combining statistics. Therefore, researchers should design and carry out trials with high selectivity, high accuracy, rigorous design and large sample size, and conduct in-depth mechanism exploration to provide a more reliable basis for the application of SGLT2i.

The major limitation of this meta-analysis is the complex and diverse population characteristics of the included studies which may induce a racial heterogeneity. Secondly, among the 49 studies, only nine RCTs and the rest trials were cohort studies, this may lead to a reduction in the methodological quality of clinical controlled studies. Furthermore, when analyzing some results, there was a significant heterogeneity and publication bias due to the small number of included studies and the complexity of population characteristics. Therefore, more prospective clinical studies with a larger sample size may strengthen the evidence.

## 5 Conclusions

In conclusion, our meta-analysis summarized the efficacy of SGLT2i in cardiovascular and cerebrovascular diseases. The incidence of cardiovascular death, myocardial infarction, heart failure and all-cause mortality was reduced with the use of SGLT2i, but no significant preventive effect was seen for the occurrence of stroke, ischemic stroke, acute coronary syndrome and revascularization. Subgroup analyses showed that the different types of SGLT2i reduced the incidence of heart failure, but only dapagliflozin reduced the incident rate of stroke. SGLT2i had a positive preventive effect on the incidence of stroke, heart failure and all-cause mortality compared to DPP-4i. Furthermore, SGLT2i significantly reduced heart failure and cardiovascular mortality in patients who were at high risk. Further, more studies focusing on the mechanism still needs to be done.

## Data Availability

The original contributions presented in the study are included in the article/**Supplementary Material**. Further inquiries can be directed to the corresponding authors.
