# Supplementary material for: Effects of sodium-glucose cotransporter 2 inhibitors on cardiovascular and cerebrovascular diseases: a meta-analysis of controlled clinical trials
Source: Front Endocrinol (Lausanne). 2024 Aug 23;15:1436217. doi: 10.3389/fendo.2024.1436217 (PMC11377240; doi:10.3389/fendo.2024.1436217)
Supplement: Supplementary file 1 [file Table1.doc]

| **Supplementary Table 1. Characteristics of the 49 included studies.** | | | | | | | | | | | | | | |
| --- | --- | --- | --- | --- | --- | --- | --- | --- | --- | --- | --- | --- | --- | --- |
| First author  Year | Study design | Country | Characteristics of participants | SGLT2i, dose | Control, dose | Number of participants, n | | Mean(±SD) age, years | | | Male (%) | | Follow-up time | Outcome index |
|  |  |  |  |  |  | SGLT2i | Control | | SGLT2i | Control | SGLT2i | Control |  |  |
| Marta Baviera  2021 | Cohort study | Lombardy, Italian | T2DM patients aged 50 years or older who have been using AHAs | SGLT2i | other AHAs except GLP-1RA or SGLT2i | 11683 | 11683 | | 68.2±7.2 | 68.8±8.3 | 62.1 | 61.5 | SGLT2i: 1.8 years^*^  Control: 1.6 years^*^ | cerebrovascular disease; all-cause mortality; ACS and HF |
|  |  | Apulia, Italian |  |  |  | 6046 | 6046 | | 67.2±7.6 | 67.33±7.9 | 55.1 | 52.8 | SGLT2i: 1.6 years^*^  Control: 1.5 years^*^ |  |
| Christopher P Cannon  2020 | RCT | multi-center study | patients 40 years of age or older with T2DM and ASCVD | Ertugliflozin  5 or 15 mg/day | Placebo | 5499 | 2747 | | 64.4±8.1 | 64.4±8.0 | 70.3 | 69.3 | 3.5 years^#^ | MACE; HF; all-cause mortality |
| Yaa-Hui Dong  2022 | Cohort study | Taiwan, China | T2DM patients aged 20 years or older who do not have high-risk conditions | SGLT2i | GLP-1RA | 13016 | 13016 | | 53.25±12.99 | 53.44±13.56 | 48.7 | 49.1 | 0.6 years^*^ | MI; stroke; HF |
| Ameenathul Mazaya Fawzy  2023 | Cohort study | multi-center study | adults with T2DM | SGLT2i | Non-SGLT2i | 131188 | 131188 | | 58.79±13.72 | 58.38±11.38 | 55.8 | 56.2 | 2 years | HF; arrhythmias; cardiac arrest; stroke; all-cause mortality |
| Edouard L Fu  2022 | Cohort study | Sweden | adult T2DM patients who are new to SGLT2i or GLP-1RA | SGLT2i | GLP-1RA | 5489 | 6886 | | 61.0±12 | 61.0±12 | 62.4 | 62.4 | 1.6 years^*^ | MACE and HF |
| Gábor Sütő  2021 | Cohort study | Hungary | adult T2DM patients who are using SGLT2i or DPP-4i | SGLT2i | DPP-4i | 18583 | 18583 | | _ | _ | _ | _ | SGLT2i: 635 days  Control: 656 days | AMI with PCI; stroke; HF; all-cause mortality |
| Antonio Gonzalez Perez  2023 | Cohort study | England | T2DM patients aged 20 to 89 years old who are using metformin | SGLT2i | Non-SGLT2i | 12978 | 44286 | | 59.6±10.2 | 60.4±10.0 | 60.9 | 62.3 | SGLT2i: 2.3 years^#^  Control: 2.1 years^#^ | MACE and all-cause mortality |
| Phyo T Htoo  2022 | Cohort study | America | T2DM patients over 65 years of age | Empagliflozin | Liraglutide | 22894 | 22894 | | 71.9±5.1 | 71.9±5.1 | 48.8 | 49.0 | 2 years | modified MACE and HF |
|  |  |  |  | Empagliflozin | Sitagliptin | 22812 | 22812 | | 72.1±5.1 | 72.1±5.1 | 53.1 | 53.0 |  |  |
| Iskandar Idris  2021 | Cohort study | England | T2DM patients with CVRD–free | SGLT2i | DPP-4i | 17353 | 17353 | | 54.8±10.89 | 54.3±12.03 | 55.3 | 55.4 | 1.9 years | MACE; HF; all-cause mortality |
|  |  |  | T2DM patients with high risk or established cardiovascular disease | SGLT2i | DPP-4i | 11367 | 11367 | | 64.3±7.24 | 64.1±8.19 | 67.5 | 66.4 | 1.8 years |  |
| Ja Young Jeon  2021 | Cohort study | Korea | adults with T2DM who are receiving metformin monotherapy | SGLT2i | DPP-4i | 21688 | 21688 | | 51.9±11.9 | 51.6±12.2 | 55.6 | 55.3 | SGLT2i: 333 days^*^  Control: 341 days^*^ | modified MACE and HF |
|  |  |  |  | SGLT2i | sulfonylurea | 20120 | 20120 | | 52.6±11.7 | 52.4±12.4 | 56.4 | 56.2 | SGLT2i: 348 days^*^  Control: 354 days^*^ |  |
| Kohei Kaku  2017 | RCT | multi-center study | T2DM patients who have CVD and eGFR≥30ml/min/1.73m^2^ | Empagliflozin  10 or 25 mg/day | Placebo | 1006 | 511 | | 61.1±9.1 | 60.7±9.4 | 74.4 | 74.2 | _ | MACE; HF; all-cause mortality |
| Mikhail Kosiborod  2018 | Cohort study | multi-center study | T2DM patients who newly treated with AHAs | SGLT2i | other AHAs | 102580 | 102580 | | _ | _ | _ | _ | SGLT2i: 254 days^#^  Control: 232 days^#^ | MI and stroke |
| Chang Hee Kwon  2022 | Cohort study | Korea | T2DM patients who were diagnosed as AF before prescription of AHAs | SGLT2i | Non-SGLT2i | 2958 | 10691 | | 58.89±12.06 | 60.02±11.91 | 59.4 | 58.6 | 2.1 years^#^ | MACE; HF; all-cause mortality |
| Hsin-Fu Lee  2020 | Cohort study | Taiwan, China | T2DM patients with concomitant peripheral artery disease | SGLT2i | DPP-4i | 11431 | 11431 | | 64.7±10.7 | 65.1±14.5 | 49.0 | 49.5 | SGLT2i: 0.96 years^#^  Control: 0.66 years^#^ | ischemic stroke; MI; HF; cardiovascular death; all-cause mortality |
| Jayoung Lim  2022 | Cohort study | Korea | T2DM patients without prior history of ASCVD, CKD or HF | Dapagliflozin | DPP-4i | 921 | 1842 | | _ | _ | 66.2 | 63.9 | 43.4 months^*^ | ACS; HF; cardiovascular death; stroke; all-cause mortality; revascularization |
|  |  |  |  | Empagliflozin |  | 921 |  |  | _ |  | 66.3 |  |  |  |
| Donna Shu-Han Lin  2022 | Cohort study | Taiwan, China | Adults with T2DM who were treated with GLP-1RA or SGLT2i | SGLT2i | GLP-1RA | 81152 | 20288 | | 56.9±13.0 | 56.4±13.2 | 51.5 | 51.6 | SGLT2i: 1.93 years^#^  Control: 1.96 years^#^ | MACE; HF; all-cause mortality |
| Tien-Hsing Chen  2020 | Cohort study | Taiwan, China | T2DM patients older than 20 years old using SGLT2i or metformin as first-line therapy | SGLT2i | Metformin | 1100 | 39920 | | 61.2±86.8 | 59.4±13.1 | 53.2 | 56.2 | 1 year | HF; ACS; ischemic stroke; all‑cause mortality. |
| Caroline H. Nørgaard  2022 | Cohort study | Denmark | patients aged 40-100 years old with T2DM using SGLT2i or GLP-1RA | SGLT2i | GLP-1RA | 5275 | 8913 | | _ | _ | 60.2 | 58.1 | 4.3 years^*^ | MACE; HF |
| Björn Pasternak  2019 | Cohort study | multi-center study | patients aged 35-84 yeras old with T2DM who are new to SGLT2i or DPP-4i | SGLT2i | DPP-4i | 20983 | 20983 | | 61.0±10 | 61.0±10 | 60.0 | 60.0 | SGLT2i: 1.1 years^*^  DPP-4i: 1.7 years^*^ | MACE; HF; all-cause mortality |
| Elisabetta Patorno  2022 | Cohort study | America | adults with T2DM | Empagliflozin | DPP-4i | 39072 | 39072 | | 60.23±9.05 | 60.32±9.23 | 54.6 | 54.5 | 6 months^#^ | MI; HF; stroke; all-cause mortality |
| Elisabetta Patorno  2018 | Cohort study | America | adults with T2DM who had not use the study drugs in the 6 months prior to the trial | Canagliflozin | DPP-4i | 17667 | 17667 | | 56.5±10.6 | 56.5±10.7 | 55.1 | 55.0 | 0.6 years^#^ | HF; MI; stroke; unstable angin; coronary revascularization; all-cause mortality |
|  |  |  |  | Canagliflozin | GLP-1RA | 20539 | 20539 | | 56.8±10.9 | 56.7±10.8 | 52.7 | 52.8 |  |  |
|  |  |  |  | Canagliflozin | Sulfonylurea | 17354 | 17354 | | 55.9±10.5 | 55.8±10.5 | 55.0 | 54.8 |  |  |
| Elisabetta Patorno  2021^a^ | Cohort study | America | adult T2DM patients with the history of cardiovascular disease | SGLT2i | GLP-1RA | 52901 | 52901 | | 67.43±9.49 | 67.44±9.50 | 56.1 | 56.2 | 7 months^*^ | hospitalization for MI or stroke; HF |
|  |  |  | adult T2DM patients without the history of cardiovascular disease | SGLT2i | GLP-1RA | 133139 | 133139 | | 58.09±11.89 | 58.11±11.89 | 45.9 | 46.1 |  |  |
| Elisabetta Patorno  2021^b^ | Cohort study | America | T2DM patients older than 65 years who are new to SGLT2i or GLP-1RA | SGLT2i | GLP-1RA | 45047 | 45047 | | 71.56±5.01 | 71.56±5.03 | 45.6 | 46.0 | 6 months^*^ | MACE; HF; all-cause mortality |
| Frederik Persson  2018 | Cohort study | multi-center study | T2DM adults who are new to dapagliflozin or DPP-4i | Dapagliflozin | DPP-4i | 10227 | 30681 | | 61±11.1 | 60.8±12.4 | 59.0 | 59.6 | 0.95 years^#^ | MACE; HF; AF; unstable angina and all-cause mortality |
| Elmor D Pineda  2020 | Cohort study | America | adults with T2DM who are new to AHAs | SGLT2i | other AHAs | 815 | 815 | | _ | _ | 51.2 | 51.2 | 1 year | MI; HF; stroke; unstable angina; revascularization |
|  |  |  |  | SGLT2i | GLP-1RA | 947 | 947 | | _ | _ | 42.3 | 42.3 |  |  |
| HoJin Shin  2022 | Cohort study | America | adults with T2DM using metformin or SGLT2i as first-line therapy | SGLT2i | Metformin | 8613 | 17226 | | 60.05±12.14 | 60.14±12.55 | 51.5 | 52.4 | SGLT2i: 10.7 months^#^  Control: 12.2 months^#^ | MI; stroke; HF; all-cause mortality |
| Reimar W Thomsen  2021 | Cohort study | Denmark | adults with T2DM who first used empagliflozin or liraglutide | Empagliflozin | Liraglutide | 14148 | 12628 | | _ | _ | 59.8 | 59.1 | 1.1 years^#^ | expanded MACE; HF; all-cause mortality; first initiation of loop-diuretic therapy |
| Stephen D Wiviott  2019 | RCT | multi-center study | patients 40 years of age or older with T2DM who had or were at risk for ASCVD | Dapagliflozin 10mg | Placebo | 8582 | 8578 | | 63.9±6.8 | 64.0±6.8 | 63.1 | 62.1 | 4.2 years^*^ | MACE; HF; all-cause mortality |
| Chun-Ting Yang  2022 | Cohort study | Taiwan, China | T2DM patients on stable medication | SGLT2i | DPP-4i | 21329 | 21329 | | 57.91±11.20 | 58.55±11.73 | 56.7 | 57.0 | 1.6 years^#^ | MACE; HF; all-cause mortality |
| Yi Zhu  2022 | Cohort study | China | T2DM patients with AMI undergoing PCI | Dapagliflozin | Non-Dapagliflozin | 141 | 645 | | 60.6±13.6 | 62.5±13.5 | 74.5 | 77.1 | 23 months^*^ | MACE; HF; all-cause mortality |
| Bernard Zinman  2017 | RCT | multi-center study | T2DM patients who have CVD and eGFR≥30ml/min/1.73m^2^ | Empagliflozin 10 or 25 mg/day | Placebo | 4687 | 2333 | | _ | _ | 71.2 | 72.0 | 3.1 years^*^ | the times of all types of strokes |
| Bernard Zinman  2015 | RCT | multi-center study | T2DM patients who have CVD and eGFR≥30ml/min/1.73m^2^ | Empagliflozin 10 or 25 mg/day | Placebo | 4687 | 2333 | | _ | _ | 71.2 | 72.0 | 3.1 years^*^ | MACE; hospitalization for unstable angina; coronary revascularization; HF; all-cause mortality |
| Sheng Chen  2020 | RCT | China | patients with T2DM at high cardiovascular risk | Canagliflozin 100mg/day | Non-Canagliflozin | 47 | 47 | | 62.18±6.89 | 64.02±6.37 | 51.1 | 51.1 | 12 months | MACE; HF; all-cause mortality |
| Xin Zhai  2020 | RCT | China | patients aged 40 years or older with T2DM at high cardiovascular risk | Canagliflozin 100mg/day | Non-Canagliflozin | 153 | 153 | | 63.9±8.3 | 63.2±7.1 | 56.9 | 58.2 | 12 months | MACE; HF; all-cause mortality |
| Yao-Hui Jiang  2021 | Cohort study | China | patients with T2DM who underwent drug-eluting stent implantation | Dapagliflozin | Non-Dapagliflozin | 522 | 1052 | | 61.25±10.6 | 62.05±9.6 | 66.9 | 61.6 | 18.7 months^#^ | MACE; all-cause mortality; stent restenosis and angina pectoris readmission |
| Yao-Hui Jiang  2022 | Cohort study | China | T2DM patients with coronary heart disease | Dapagliflozin | Non-Dapagliflozin | 671 | 1310 | | 61.87±10.7 | 62.75±10.1 | 65.0 | 61.3 | 12.7 months^#^ | MACE; all-cause mortality; revascularization and angina pectoris readmission |
| Chun-Xia Li  2019 | RCT | China | T2DM patients | Canagliflozin | Non-Canagliflozin | 78 | 78 | | 47.52±11.05 | 47.61±11.13 | 60.3 | 59.0 | 3 months | stroke |
| Yan-Ping Yin  2021 | Cohort study | China | T2DM patients who had an AMI and were taking metformin | Dapagliflozin | Non-Dapagliflozin | 30 | 30 | | 47.51±12.57 | 48.03±11.69 | 53.3 | 50.0 | 1 month | revascularization; stroke; recurrence of MI |
| Elvira D'Andrea  2023 | Cohort study | America | T2DM patients | SGLT2i | DPP-4i | 60523 | 84091 | | 59.4±11.6 | 64.0±12.1 | 55.8 | 50.6 | 240 days | modified MACE and HF |
| Wei-Syun Hu  2023 | Cohort study | Taiwan, China | patients 20 years of age or older with heart failure | SGLT2i | Non-SGLT2i | 17588 | 17588 | | 64.96±12.43 | 65.29±12.35 | 58.3 | 57.98 | SGLT2i: 1.75 years^#^  Control: 1.99 years^#^ | MACE and all-cause mortality |
| Hui-Jeong Hwang  2023 | Cohort study | Korea | T2DM patients who were newly diagnosed with cancer and treated with anthracycline-containing chemotherapy | SGLT2i | Non-SGLT2i | 779 | 2337 | | 56±10 | 56±10 | 28.6 | 27.1 | 3.4 years^#^ | HF; AMI; ischemic stroke; all-cause mortality |
| Alexander Kutz  2023 | Cohort study | America | patients 65 years of age or older with T2DM who have not used the study drugs in one year | SGLT2i | DPP-4i | 120202 | 120202 | | 72.28±5.38 | 72.27±5.51 | 52.3 | 51.9 | 10.6 months^#^ | AMI; ischemic stroke; HF; all-cause mortality |
|  |  |  |  | SGLT2i | GLP-1RA | 89865 | 89865 | | 71.79±5.14 | 71.83±5.20 | 48.8 | 49.0 | 9.6 months^#^ |  |
| Osung Kwon  2023 | Cohort study | Korea | T2DM patients who received SGLT2i continuously for 7 days after PCI | SGLT2i | Non-SGLT2i | 938 | 1876 | | 56.4±11.3 | 57. 6 ±11. 3 | 82.0 | 79.0 | 2.1 years^*^ | MI; ischemic stroke; HF; all-cause mortality |
| Hsin-Fu Lee  2023 | Cohort study | Taiwan, China | T2DM patients who received study drugs continuously after PCI | SGLT2i | DPP-4i | 4110 | 4110 | | 61.7±11.3 | 62.3±10.8 | 78.7 | 79.4 | SGLT2i: 1.73 years^#^  Control: 1.69 years^#^ | MI; HF; ischemic stroke; revascularization; all-cause mortality. |
| Young Sang Lyu  2023 | Cohort study | Korea | T2DM patients who received metformin and study drugs after AMI | SGLT2i | DPP-4i | 186 | 593 | | 59.11±11.52 | 66.12±10.86 | 80.7 | 71.2 | 12 months | MACE; all-cause mortality; revascularization; rehospitalization |
| Natalie McCormick  2023 | Cohort study | British Columbia,  Canada | adult T2DM patients with gout | SGLT2i | DPP-4i | 4075 | 4075 | | 66.04±10.79 | 66.03±12.08 | 71.5 | 71.2 | 1.6 years^#^ | MI and stroke |
| Tadarro L Richardson Jr  2023 | Cohort study | America | Veterans 18 years of age or older with T2DM | SGLT2i | DPP-4i | 21170 | 21170 | | _ | _ | 94.2 | 94.1 | SGLT2i: 0.42 years^*^  Control: 0.47 years^*^ | MACE and HF |
| Ying-Ying Liu  2023 | Cohort study | China | T2DM patients aged 60 to 85 years old who underwent bioprosthetic valve replacement | Canagliflozin | Non-SGLT2i | 112 | 112 | | 72.3±8.4 | 67.9±9.5 | _ | _ | 13.5 months^#^ | cardiovascular death or cardiac arrest; stroke; HF; ACS |
| Jian Yang  2022 | RCT | China | T2DM patients aged 45-75 years old with acute chest pain who received metformin | Dapagliflozin | Non-Dapagliflozin | 51 | 51 | | 57.26±10.35 | 59.47±9.54 | 54.9 | 47.1 | 1 month | cardiovascular death; recurrence of MI; stroke; revascularization; HF |

* The median follow-up time

# The mean follow-up time

Footnotes: T2DM, type 2 diabetes mellitus; AHA, antihyperglycemic agent; SGLT2i, sodium-glucose cotransporter 2 inhibitor; GLP-1RA, glucagon-like peptide 1; ACS, acute coronary syndrome; HF, heart failure; RCT, randomized controlled trial; ASCVD, arteriosclerotic cardiovascular disease; MACE, major adverse cardiovascular events; MI, Myocardial infarction; DPP-4i, dipeptidyl peptidase 4 inhibitor; AMI, acute myocardial infarction; PCI, percutaneous coronary intervention; CVRD, cardiovascular and renal disease; CVD, Cardiovascular disease; eGFR, Estimated glomerular filtration rate; AF, atrial fibrillation; CKD, Chronic kidney disease.
